# Fungal Infections and Colonization after Bilateral Lung Transplant: A Six-Year Single-Center Experience

**DOI:** 10.3390/jof10010080

**Published:** 2024-01-19

**Authors:** Annalisa Boscolo, Annamaria Cattelan, Serena Marinello, Francesca Medici, Giovanni Pettenon, Sabrina Congedi, Nicolò Sella, Nicolò Presa, Elisa Pistollato, Stefano Silvestrin, Martina Biscaro, Luisa Muraro, Arianna Peralta, Maria Mazzitelli, Andrea Dell’Amore, Federico Rea, Paolo Navalesi

**Affiliations:** 1 Department of Medicine, University of Padua, 35122 Padua, Italy; annalisa.boscolo@gmail.com (A.B.); francesca.medici.1@studenti.unipd.it (F.M.); sabrina.congedi@studenti.unipd.it (S.C.); elisa.pistollato@gmail.com (E.P.); martina.biscaro@studenti.unipd.it (M.B.); paolo.navalesi@unipd.it (P.N.); 2 Anesthesia and Intensive Care Unit, Padua University Hospital, 35128 Padua, Italyluisa.muraro@aopd.veneto.it (L.M.); arianna.peralta@aopd.veneto.it (A.P.); 3 Department of Cardiac, Thoracic, Vascular Sciences, and Public Health, University of Padova, 35122 Padua, Italy; stefano.silvestrin@aopd.veneto.it (S.S.); andrea.dellamore@unipd.it (A.D.); federico.rea@unipd.it (F.R.); 4 Infectious and Tropical Diseases Unit, Padua University Hospital, 35128 Padua, Italy; annamaria.cattelan@unipd.it (A.C.); serena.marinello@aopd.veneto.it (S.M.); nicolo.presa@aopd.veneto.it (N.P.)

**Keywords:** invasive fungal infections, fungal colonization, solid organ transplant, lung transplant, bilateral lung transplant, prophylaxis

## Abstract

Fungal infections (FIs) are one of the leading causes of morbidity and mortality within the first year of lung transplant (LT) in LT recipients (LTRs). Their prompt identification and treatment are crucial for a favorable LTR outcome. The objectives of our study were to assess (i) the FI incidence and colonization during the first year after a bilateral LT, (ii) the risk factors associated with FI and colonization, and (iii) the differences in fungal incidence according to the different prophylactic strategies. All bilateral LTRs admitted to the intensive care unit of Padua University Hospital were retrospectively screened, excluding patients <18 years of age, those who had been re-transplanted, and those who had received ventilation and/or extracorporeal membrane oxygenation before LT. Overall, 157 patients were included. A total of 13 (8%) patients developed FI, and 36 (23%) developed colonization, which was mostly due to *Aspergillus* spp. We did not identify independent risk factors for FI. Groups of patients receiving different prophylactic strategies reported a similar incidence of both FI and colonization. The incidence of FI and fungal colonization was 8% and 23%, respectively, with no differences between different antifungal prophylaxes or identified predisposing factors. Further studies with larger numbers are needed to confirm our results.

## 1. Introduction

Since the first European Organization for Research and Treatment of Cancer/Invasive Fungal Infections Cooperative Group (EORTC) proposal in 2002 [1], several efforts have been made to clarify and standardize both the definitions and the diagnostic criteria of fungal infection (FI). According to the most recent Guidelines published in 2017 by the European Society for Clinical Microbiology and Infectious Diseases (ESCMID) [2], the diagnosis of a FI is based on radiological imaging, clinical findings, and serological and microbiological tests. Moreover, the European Organization for Research and Treatment of Cancer and the Mycoses Study Group Education and Research Consortium classify the diagnosis of FI as proven (i.e., histopathologic or mycological evidence of a fungus from a normally sterile site and radiological and clinical signs of infection), probable (i.e., the presence of one clinical factor plus mycologic evidence), or possible (i.e., either clinical or mycological evidence) [3].

FIs are a very common complication in immunocompromised individuals, particularly in those receiving a solid organ transplant (SOT). Moreover, the type of SOT, the time from transplant, the presence of bacterial, viral, or fungal colonization before transplant, and the immunosuppressive regimen are factors influencing the risk of developing a postoperative FI [4,5,6,7]. FIs appear to be one of the most significant concerns in lung transplant recipients (LTRs), representing a substantial threat to the long-term success of the transplant, with a recognized association between fungal infections and bronchiolitis obliterans syndrome [8,9].

Factors potentially predisposing patients to develop FIs after LT are (i) rejection and high levels of pharmacologic immunosuppression, (ii) constant exposure to the environment, which allows direct access of fungal pathogens to the allograft, (iii) a reduction in mucociliary clearance and altered macrophage phagocytic function, (iv) structural abnormalities that predispose the host to develop colonization, (v) early ischemia of bronchial anastomosis, (vi) single-LT, (vii) hypogammaglobulinemia (IgG < 400 mg/dL), (viii) *Cytomegalovirus* (CMV) infection, and (ix) *Aspergillus* spp. colonization within a year [9,10].

According to the Transplant-Associated Infection Surveillance Network (TRANSNET), the cumulative incidence of FI one year after LT was estimated to be around 8.6% [9,11,12]. The overall median time from transplant to the FI onset was reported to be up to 11 months [6,7,13]. *Aspergillus* and *Candida* species are the most isolated species, accounting for 44% and 23% of FIs, respectively [5]. Although candidiasis usually occurs early (after a median of 1 month after LT), often causing candidemia and pleural space, chest wall, or chest wound infections, infections by *Aspergillus* spp. seems to occur later, during the first 6 months, with a median time of 3.2 months after LT, frequently causing pulmonary diseases, such as ulcerative pneumonia, tracheobronchitis, bronchial anastomotic infection, or even disseminated disease [6,14].

Considering fungal colonization, as per the definitions given in [3], the true incidence before and after LT is still a matter of debate. Some authors report an incidence preceding the LT ranging between 59% and 70% [12,13], while one reports an incidence in a post-LT range between 8% and 16% [12], with the most recently reported prevalence being between 10% and 12% [9,10,11,12,13,14,15]. It is reasonable to think that the impacts of fungal infections and colonizations on LT outcomes are quite different. Indeed, patients with FIs have a higher risk of being re-hospitalized, being re-intubated, and requiring a longer period of O_2_ therapy when compared to patients who have just a fungal colonization [12,13]. Obviously, all these assumptions underline the importance of performing strict microbiological surveillance for the prevention of FI and for the minimization of fungal colonization risk.

Antifungal prophylaxis is the most common strategy to prevent FIs after LT, and three different strategies have been proposed: (i) universal prophylaxis (to be given to all patients immediately after LT), (ii) targeted prophylaxis (in patients with known risk factors), and (iii) pre-emptive prophylaxis (in patients who are colonized but without evidence of an ongoing fungal disease) [8]. To date, no consensus has been reached about the best approach (i.e., universal versus targeted versus pre-emptive versus none) or the type and duration of antifungal therapy [11,16,17,18,19]. Based on recent descriptive data, and even though solid evidence is still lacking, most transplant centers (about 90% in the US in 2019) prefer to adopt the universal prophylaxis strategy [19]. However, data about potential differences in clinical responses after antifungal therapy in pre-colonized LT recipients experiencing FI, as well as data about the comparison between the latter and those without pre-existing fungal isolations, are still lacking. However, further investigations are necessary to confirm these preliminary data.

Therefore, we designed this single-center retrospective study including bilateral LTRs undergoing universal prophylaxis, with the aim of assessing (i) the 1-year incidence of FI and colonization, (ii) potential risk factors for FI, and (iii) differences in terms of the incidence of FI or colonization, according to different prophylaxes.

## 2. Materials and Methods

All adult patients admitted to our Intensive Care Unit (ICU) at Padua University Hospital after their first bilateral LT between 1 February 2016 and 1 October 2022 were retrospectively evaluated. Exclusion criteria were: (i) age under 18 years; (ii) single or newer LT; (iii) invasive mechanical ventilation (IMV), veno–venous (V–V), or veno–arterial (V–A) extracorporeal membrane oxygenation (ECMO) before lung transplant.

The study was approved by the Ethics Committee of Padua University Hospital (reference n. 0054869, 4539/AO/18) and conducted in accordance with the principles of Good Clinical Practice and the Declaration of Helsinki. Patient consent was waived due to the retrospective nature of the study, as per Italian law (Italian Drug Agency note, 20 March 2008, No. 76). The article was written in accordance with the Strengthening the Reporting of Observational Studies in Epidemiology (STROBE) checklist [20]. Standardized protocols for perioperative antifungal management and immunosuppressive therapy have been developed in our center following international recommendations [21,22]. Specifically, standard perioperative antifungal prophylaxis provides systemic liposomal amphotericin B (3 mg/Kg) plus nebulized lipid amphotericin B (25 mg daily) therapy until December 2019, and only systemic liposomal amphotericin B, without inhalation therapy, from December 2019 onwards. In selected patients, different perioperative prophylaxis regimens were used, according to the previously known preoperative colonization.

After hospital discharge, antifungal prophylaxis was administered using azoles for at least 3 months. In the case of diagnosed postoperative infections, targeted antifungal therapy was prescribed [2,3,15,18,23]. Regarding immunosuppressive treatment, an IV dose of methylprednisolone was administered before each graft reperfusion during the surgical procedure, followed by induction with basiliximab 20 mg/day on postoperative days 0 and 4. Maintenance therapy was the following: (i) cyclosporine, with a target plasma concentration of 250 ng/mL, from February 2016 through December 2019; or (ii) tacrolimus, with a target plasma concentration of 10–15 ng/mL, after December 2019. Furthermore, mycophenolate mofetil was provided from 1 to 2 g/day, and methylprednisolone was provided at the dosage of 0.5 mg/kg/day [24]. In our center, we implemented a standardized protocol for microbiological surveillance based on a routine bi-weekly or thrice-weekly collection of respiratory and rectal samples from ICU admission to hospital discharge, as previously published [24,25,26]. Additionally, biological fluid samples for microbiological purposes were collected from donors [24]. Moreover, a further diagnostic workup is implemented based on specific syndrome-related features. For each patient recruited, baseline characteristics, underlying disease, previous donor-related or recipient-related colonization, surgical characteristics, antifungal therapies, and outcomes of interest (i.e., re-tracheal intubation and/or tracheostomy, invasive mechanical ventilation, 30-day rejection, ICU length of stay (LOS), hospital LOS, hospital and 1-year mortality) were collected from electronic health records. Patients were divided into 3 groups: the FI group (i), including patients with the presence of fungus in respiratory secretions (i.e., bronchoalveolar lavage (BAL)) detected by culture, polymerase chain reaction (PCR) or biomarkers (i.e., galactomannan (GM) antigen) in the presence of symptoms, radiological and endobronchial changes, or presence of histological changes consistent with fungal invasion of tissue [3,8]; the group of ‘fungal colonization’ (ii), when mycological evidence was not supported by clinical symptoms, radiological or endobronchial changes; and all the ‘others’ (iii) including the population without evidence of FIs or colonization.

Patient baseline characteristics were summarized through descriptive statistics: categorical variables were expressed as numbers and percentages, and continuous variables were reported as median and interquartile range (IQR), as appropriate. No imputation for missing data was planned. The Kruskal–Wallis H test, a rank-based nonparametric test, has been used to determine whether there are statistically significant differences between the three subpopulations. The Wilcoxon signed-rank test was used for the comparison of variables between three groups (“FI” group versus “fungal colonization” group versus others). A multivariable logistic regression model has been used to identify potential independent risk factors for FI. The independent predictors have been identified through a stepwise regression approach. This approach combines forward and backward selection methods in an iterative procedure (with a significance level of 0.05 both for entry and retention) to select predictors in the final multivariable model. Independent variables used in the stepwise approach were septic end-stage lung disease, recipient-related virus, surgical revisions, and prolonged ECMO. The Kaplan–Meier curve was used to estimate the cumulative probability of 1-year survival in the three subgroups. The level of significance was established at *p* < 0.05. All analyses were performed with R version 4.0.3 (R Foundation for Statistical Computing, Vienna, Austria) and Prism (version 5.0; GraphPad Software, Inc., La Jolla, CA, USA).

## 3. Results

**Figure 1:**
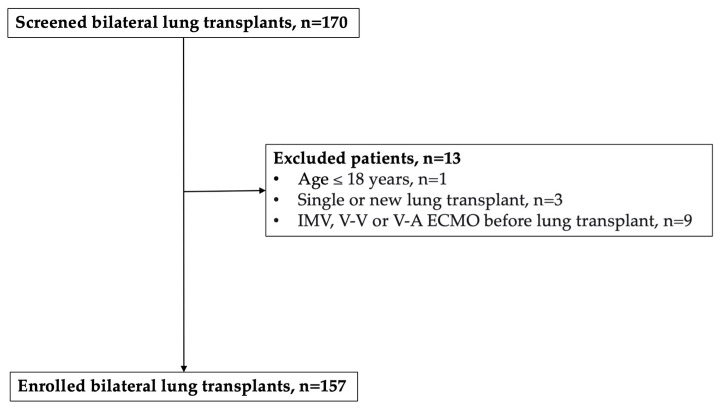
Study flow. Abbreviations: IMV, invasive mechanical ventilation; V–V, veno–venous; V–A, veno–arterial; ECMO, extracorporeal membrane oxygenation.

Over the study period, 170 patients who received bilateral LT were screened, and 13 subjects were excluded according to exclusion criteria, thus including 157 patients (see study flow, Figure 1). They were mostly males (65%), with a median age of 54 years (IQR 41–60), with no epidemiological or demographical significant differences among the three groups (Table 1 and Table 2). Among 157 patients, 13 (8%) developed FI within 1 year of LT, while 36 (23%) became colonized by fungal species (either *Aspergillus* or *Candida* spp.). Regarding FIs, Aspergillus spp. accounted for most infections (10, 77%), which occurred in the respiratory tract in all cases, while no fungal species were ever isolated from blood cultures (Table 3). The median time from LT to Aspergillus spp. and Candida spp. isolation was 110 days (IQR 27–165) and 12 days (IQR 4–34), respectively (Table 3).

Regarding patients’ baseline characteristics, both pre-transplant recipient-related bacterial and viral colonizations were more frequent in patients who developed fungal infection and colonization (*p* = 0.045 and 0.003, respectively) compared to those who did not, while previous fungal recipient- or donor-related colonization were not statistically different among the three subpopulations (Table 1).

However, according to the multivariable analysis, neither previous recipient-related bacterial or viral isolations were identified as an independent risk factor for FI in our cohort (*p* = 0.78 and 0.52, respectively) (Table 1).

Regarding the postoperative antifungal regimen, most patients (112, 71%) received prophylactic therapy based on intravenously given liposomal amphotericin B plus an inhaled amphotericin B lipid complex (Table 2 and Table 3). The overall length of prophylaxis was 271 (IQR: 169–479) days, and the incidence of FIs in this group was 8% (*n* = 9). No differences in terms of FI or colonization were found considering different prophylactic regimens (Table 3).

Considering FI biomarkers (Table 2), high values of the GM index dosed on BAL were significantly associated with a diagnosis of FI (*p* < 0.001), while no differences in β-D-glucan levels (reference > 80 pg/mL) were observed between patients with FIs and fungal colonization (*p* = 0.72). Interestingly, only 6 (46%) patients with FI and 6 (17%) with fungal colonization recorded a GM index above 1 (*p* = 0.17) (Table 2).

**Figure 2:**
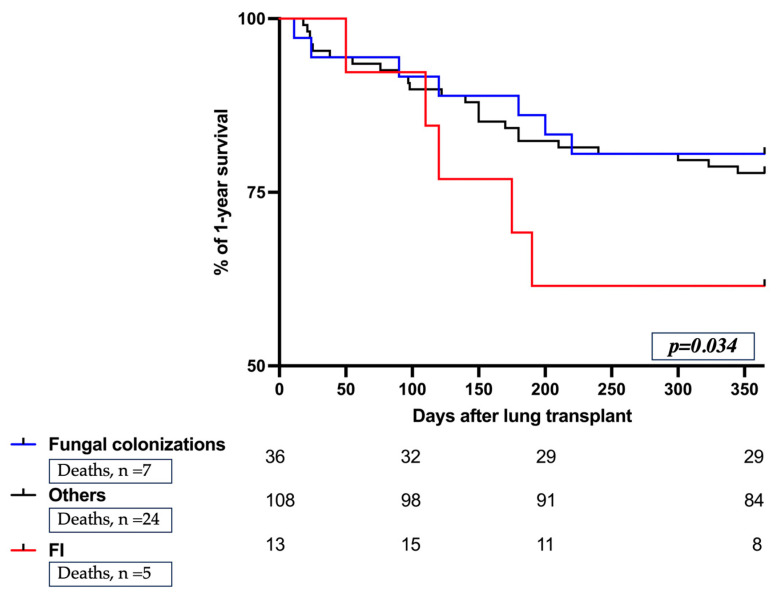
1-year mortality distribution among the different study groups. Kaplan–Meier curves estimate the cumulative probability of 1-year survival from lung transplant. Follow-up was conducted from lung transplant up to 1 year. The numbers of patients remaining in the study are reported below the curves. Abbreviations: FI, Fungal Infection; n, number.

Interestingly, patients who developed FIs required re-intubation and/or tracheostomy more frequently compared to the other recipients (*p* = 0.007) (Table 4); and, finally, we observed a trend towards a lower survival in the FI group compared to the other groups, no statistically significant differences were detected in the 1-year mortality distribution between different subgroups (Table 4 and Figure 2).

## 4. Discussion

This single-center retrospective analysis analyzes risk factors linked to FIs and fungal colonization in LTRs. Our results are very similar in terms of prevalence to those described in similar past experiences and meta-analyses, but considering the high number of cases we included, we believe that it may have a clinical value.

Our study showed that, within the first year after bilateral LT, the incidence of FI was 8%, for which *Aspergillus* spp. was the main cause, while 23% of patients developed fungal colonization. Despite previous recipient-related bacterial and viral isolation often occurring in the case of fungal isolation, neither characteristic was an independent risk factor for FI. Moreover, no differences were found in terms of the incidence of FI or colonization according to different universal antifungal prophylaxis.

A similar study [14] showed FIs and colonization prevalence of 7.0% and 4.8%, respectively. Although the prevalence of FIs is like that reported in this study, the prevalence of colonization we detected is far higher, and it may be explained by at least two reasons. The first one relies on different patient’s features. Indeed, while we include all patients who received a BLT, Pennington et al. included patients with known chronic lung allograft dysfunction (CLAD) and who were free from fungi (either colonization or episode of infections) within 1 year prior to CLAD onset [13]. The second one could be related to a different strategy implemented in regular mycological monitoring and surveillance, which was stricter and more systematically performed in our study. In this regard, even if it may be argued that colonization itself is not related to infection in most cases, we believe that strict microbiological monitoring and surveillance could help clinicians identify and treat infections, eventually improving patient clinical outcomes. Moreover, careful integrated assessment with infectious disease specialists is also required to avoid treatment when it is not necessary.

Due to the remarkable worldwide increase of SOT, we feel that more data are required on this topic, especially on the impact of colonization on the short and long-term clinical outcomes of BLTRs. According to the most recent data with which ours are in line, the incidence of FI in LT ranged from 3% up to 19% [5,9]. Also, regarding fungal species, *Aspergillus* and *Candida* spp. have been confirmed as the most common fungal pathogens [9], but with a slightly different detection timeline according to the fungal species (later for *Aspergillus* spp., earlier for *Candida* spp.) and according to what has previously been reported [8,10,13]. Considering the subset of patients with fungal colonization, as mentioned, the real incidence, before and after LT, is still conflicting. Recently, some authors reported a post-LT fungal colonization incidence ranging between 10% and 12% [9,10,11,12,13,14,15], barely lower than ours (23%). The reasons behind these differences could be related to a more systematic and targeted microbiological surveillance after LT and a prompt administration of fungal prophylaxis in the overall study cohort. Under these circumstances, the overall incidence of FI appears to be relatively lower, while the incidence of fungal colonization is barely higher than previous findings [9,10,11,12,13,14,15].

Regarding the potential differences in baseline patients’ characteristics among the different three groups, previous viral and bacterial infections were more frequently recorded in the case of fungal isolation, probably because the concomitant presence of CMV or bacteria-predisposed LT recipients to an additional immunosuppression [27,28,29,30]. Furthermore, our findings suggest that the need for re-intubation and/or tracheostomy was associated with a higher rate of FI diagnosis. In fact, the need for re-intubation, which means failure weaning from invasive mechanical ventilation, has been shown to predispose to an increased occurrence of FIs in critically ill patients [31,32]. It is also to be specified that the development of FIs could be related to these two unfavorable outcomes. However, there are no studies exclusively focused on bilateral LT recipients, as we did.

Regarding antifungal preventative strategies, the antifungal prophylaxis protocol was changed in our center in December 2019 by removing inhaled amphotericin B lipid complex and maintaining only the intravenous liposomal amphotericin B administration. By comparing LTR outcomes between different prophylaxis before and after December 2019, we did not find any statistically significant differences in terms of incidence of FI or colonization (see Table 3). Despite the small sample size we recruited, this finding may suggest that the inhaled amphotericin B lipid complex, despite its safety and tolerability [33], does not seem to provide additional advantages in FI prevention.

Furthermore, no statistical differences were found in terms of 1-year mortality in the three study subpopulations. However, our mortality rate in the FI group was 38%, remarkably lower than the rates previously published (ranging between 52% and 55%), probably due to constant infection control procedures and fungal surveillance from ICU admission until one year after LT [34].

In terms of microbiological tools, the detection of BAL GM antigen using an enzyme immunoassay (EIA) became the ‘gold standard’ test for the diagnosis of Aspergillosis [35,36]. In our study, we found a statistically significant association between an elevated BAL GM index and the diagnosis of FIs, supporting its role as a valid diagnostic tool also in this specific sub-population.

Indeed, the application of advanced mycological screening for prompt diagnosis and dedicated treatments, based on drug susceptibility testing when available, is recommended for improving survival after LT [37].

This study is somewhat limited by its retrospective nature, by a relatively low number of enrolled patients, and by the different prophylactic strategies implemented over time. Lastly, no long-term follow-up is available, and the onset of potential adverse effects under different antifungal prophylaxis has never been investigated [38].

## 5. Conclusions

In our cohort of 157 bilateral LT recipients, the incidence of FI at 1 year was 8%, and *Aspergillus* spp. was the most frequently detected agent, accounting for more than 70% of invasive cases reported, while the incidence of fungal colonization was 23%. Previous recipient-related bacterial and viral isolation often occurred in the case of fungal isolation, but neither characteristic was an independent risk factor for FI. Finally, no differences, in terms of fungal isolation, were found considering different antifungal prophylaxis.

## Figures and Tables

**Table 1:** Patients’ characteristics.

Overall (*n* = 157, 100%)		FI (*n* = 13, 8%)	Fungal Colonization (*n* = 36, 23%)	Others (*n* = 108, 69%)	*p*-Value	Multivariable Analysis OR [95% CI], *p*-Value
Baseline characteristics						
Age, years, median (IQR)	54 (41–60)	50 (35.5–57.5)	48 (36.75–60.25)	55 (46–61)	0.19	-
Sex, male, *n* (%)	102 (65)	8 (61)	24 (67)	70 (65)	0.94	-
BMI, kg/m^2^, median (IQR)	24 (20–26)	25 (24–25)	22 (18–28)	23 (18–27)	0.61	-
Patients on Corticosteroids, *n* (%)	84 (54)	5 (38)	21 (58)	58 (54)	0.48	-
Patients on O_2_ therapy, *n* (%)	141 (90)	11 (85)	33 (92)	97 (90)	0.77	-
Diabetes, *n* (%)	28 (19)	1 (7)	5 (14)	22 (20)	0.41	-
Oto score, median (IQR)	5 (2–44)	4 (1–6)	5 (2–24)	5 (2–50)	0.35	-
LAS score, median (IQR)	34 (32–38)	38 (38–38)	34 (31–34)	33 (26–36)	0.20	-
Time from admission, days, *n* (%)	147 (94)	11 (85)	34 (94)	102 (94)	0.38	-
**Underlying disease**						
Septal ^a^, *n* (%)	31 (19)	3 (23)	12 (33)	16 (15)	0.05	-
Interstitial ^b^, *n* (%)	61 (39)	4 (31)	11 (31)	46 (43)	0.36	-
Obstructive ^c^, *n* (%)	23 (15)	0 (0)	6 (17)	17 (16)	0.29	-
Others ^d^, *n* (%)	42 (27)	6 (46)	7 (19)	29 (27)	0.18	-
**Previous colonization**						
Recipient-related bacteria, *n* (%)	41 (26)	4 (31)	11 (31)	26 (24)	**0.045**	2.23 [0.03–7], *p* = 0.78
Recipient-related fungi, *n* (%)	7 (5)	2 (15)	2 (6)	3 (3)	0.19	-
Recipient-related virus, *n* (%)	35 (22)	4 (31)	12 (33)	19 (18)	**0.003**	2.48 [1.05–4.61], *p =* 0.52
Donor-related bacteria, *n* (%)	77 (49)	6 (46)	17 (47)	54 (50)	0.83	-
Donor-related fungi, *n* (%)	20 (13)	1 (8)	7 (19)	12 (11)	0.22	-

Data are expressed as number and (percentage) or median and [interquartile range or IQR]. ^a^ Septic: cystic fibrosis, bronchiectasis; ^b^ Interstitial: idiopathic pulmonary fibrosis, allergic extrinsic alveolitis, non-specific interstitial pneumonia, fibrosing emphysema, lymphocytic interstitial pneumonia, respiratory bronchiolitis interstitial lung; ^c^ Obstructive: chronic obstructive pulmonary disease; ^d^ Others: idiopathic pulmonary hypertension, veno-occlusive disease, connective tissue disease, α1-anti-trypsin deficiency, lymphangioleiomyomatosis, histiocytosis, sarcoidosis, graft versus host disease. Abbreviations: FI Fungal Infection, BMI, body mass index; *n*, number; O_2_, oxygen; LAS, lung allocation score; OR, odds ratio; CI, confidential interval; IQR, interquartile range.

**Table 2:** Surgical characteristics and fungal prophylaxis.

Overall (*n* = 157, 100%)		FI (*n* = 13, 8%)	Fungal Colonization (*n* = 36, 23%)	Others (*n* = 108, 69%)	*p*-Value
Surgical characteristics					
Time from LT, days, median (IQR)	420 (365–480)	430 (382–450)	435 (378–502)	420 (359–481)	0.64
Time of graft ischemia, days, median (IQR)	560 (441–655)	563 (461–731)	575 (433–696)	553 (452–630)	0.51
Intraoperative ECMO, *n* (%)	103 (66)	8 (62)	26 (72)	77 (71)	0.75
Surgical revision, *n* (%)	29 (18)	4 (31)	10 (28)	15 (14)	0.09
Prolonged ECMO, *n* (%)	29 (18)	5 (38)	8 (22)	16 (15)	0.22
Duration of prolonged ECMO, days, median (IQR)	2 (0–3)	3 (1–8.75)	3 (2–4)	1 (0–3)	0.79
**During ICU stay**					
Immunosuppressive therapy ^+^, *n* (%)	93 (59)	9 (69)	24 (67)	60 (56)	0.50
Renal replacement therapy, *n* (%)	24 (17)	3 (23)	8 (22)	13 (12)	0.30
Antifungal therapies * and biomarkers					
Universal prophylaxis, *n* (%)					
(i) Liposomal amphotericin B i.v. plus inhaled amphotericin B lipid complex	112 (71)	9 (69)	25 (69)	78 (72)	0.79
(ii) Liposomal amphotericin B i.v.	33 (21)	2 (15)	6 (17)	25 (23)	0.79
(iii) Azoles or echinocandins	12 (8)	2 (15)	5 (14)	5 (5)	0.79
Mycological culture, *n* (%)	49 (31)	13 (100)	36 (100)	-	0.99
BAL galactomannan antigen, ratio	0 [0–3]	4 [1–6]	0 [0–2]	-	**<0.001**
BAL galactomannan antigen > b1, *n* (%)	12 (8)	6 (46)	6 (17)	-	0.17
β-D-glucan (reference > 80 pg/mL), *n* (%)	8 (5)	3 (23)	5 (14)	-	0.72

Data are expressed as number and (percentage) or median and [interquartile range]. *: additional information is available in Table 3. Abbreviations: FI, Fungal Infection; LT, lung transplant; *n*, number; ECMO, extracorporeal membrane oxygenation; i.v., intravenous; min, minutes; BAL, bronco-alveolar lavage; OR, odds ratio; IQR, interquartile range. ^+^ Immunosuppressive therapy was cyclosporine as reference.

**Table 3:** Additional details on perioperative antifungal prophylaxis.

	Liposomal Amphotericin B iv. Plus Inhaled Amphotericin B Lipid Complex (*n* = 112, 71%)	Liposomal Amphotericin B iv. (*n* = 33, 21%)	Azoles or Echinocandins (*n* = 12, 8%)	*p*-Value
Antifungal therapies and biomarkers				
FI, *n* (%)	9 (8)	2 (6)	2 (17)	0.29
Fungal colonization, *n* (%)	25 (22)	6 (16)	5 (42)	0.29
Others, *n* (%)	78 (70)	25 (76)	5 (42)	0.29

Abbreviations: n, numbers; FI, fungal infection; i.v., intravenous; min, minutes; BAL, bronco-alveolar lavage.

**Table 4:** Study outcomes.

Overall (*n* = 157, 100%)		FI (*n* = 13, 8%)	Fungal Colonization (*n* = 36, 23%)	Others (*n* = 108, 69%)	*p*-Value
Primary outcome					
1-year fungal isolation, *n* (%)					
(i) Aspergillus	26 (17)	10 (77)	16 (44)	-	**0.013**
(ii) Candida albicans	16 (10)	0 (0)	16 (44)	-	**0.013**
(iii) Others	7 (5)	3 (23)	4 (11)	-	**0.013**
Time of fungal isolation, days, median (IQR)	65 (12–142)	34 (8–143)	80 (12–142)	-	0.48
(i) Aspergillus	110 (27–165)	35 (11–135)	122 (53–203)	-	0.18
(ii) Candida albicans	12 (4–34)	-	11 (3–34)	-	0.18
**Other outcomes**					
Re-tracheal intubation and/or tracheostomy, *n* (%)	37 (24)	7 (54)	10 (28)	20 (19)	**0.007**
Invasive mechanical ventilation, hours, median (IQR)	36 (21–73)	23 (10–180)	42 (19–150)	36 (22–93)	0.97
30-day rejection, *n* (%)	27 (17)	4 (31)	5 (14)	18 (17)	0.67
ICU LOS, days, median (IQR)	8 (6–16)	5 [9–25]	5 (10–26)	8 (5–16)	0.29
Hospital LOS, days, median (IQR)	33 (29–45)	40 (29–79)	35 (30–59)	32 (28–43)	0.14
Hospital mortality, *n* (%)	13 (8)	2 (15)	2 (6)	8 (7)	0.51
1-year mortality, *n* (%)	36 (23)	5 (38)	7 (19)	24 (22)	0.64

Data are expressed as number and (percentage) or median and [interquartile range]. Abbreviations: FI, Fungal Infection; *n*, number; ECMO, extracorporeal membrane oxygenation; ICU, intensive care unit; IQR, interquartile range; LOS, length of stay.

## Data Availability

Data are contained within the article.
